# A Review of Traditional and Novel Treatments for Seizures in Autism Spectrum Disorder: Findings from a Systematic Review and Expert Panel

**DOI:** 10.3389/fpubh.2013.00031

**Published:** 2013-09-13

**Authors:** Richard E. Frye, Daniel Rossignol, Manuel F. Casanova, Gregory L. Brown, Victoria Martin, Stephen Edelson, Robert Coben, Jeffrey Lewine, John C. Slattery, Chrystal Lau, Paul Hardy, S. Hossein Fatemi, Timothy D. Folsom, Derrick MacFabe, James B. Adams

**Affiliations:** ^1^Arkansas Children’s Hospital Research Institute, Little Rock, AR, USA; ^2^Rossignol Medical Center, Irvine, CA, USA; ^3^University of Louisville, Louisville, KY, USA; ^4^Autism Recovery and Comprehensive Health Medical Center, Franklin, WI, USA; ^5^Autism Research Institute, San Diego, CA, USA; ^6^New York University Brain Research Laboratory, New York, NY, USA; ^7^MIND Research Network, University of New Mexico, Albuquerque, NM, USA; ^8^Hardy Healthcare Associates, Hingham, MA, USA; ^9^University of Minnesota Medical School, Minneapolis, MN, USA; ^10^University of Western Ontario, London, ON, Canada; ^11^Arizona State University, Tempe, AZ, USA

**Keywords:** anti-epileptic drugs, autism spectrum disorder, epilepsy, gluten-free casein-free diet, ketogenic diet, seizures, treatment

## Abstract

Despite the fact that seizures are commonly associated with autism spectrum disorder (ASD), the effectiveness of treatments for seizures has not been well studied in individuals with ASD. This manuscript reviews both traditional and novel treatments for seizures associated with ASD. Studies were selected by systematically searching major electronic databases and by a panel of experts that treat ASD individuals. Only a few anti-epileptic drugs (AEDs) have undergone carefully controlled trials in ASD, but these trials examined outcomes other than seizures. Several lines of evidence point to valproate, lamotrigine, and levetiracetam as the most effective and tolerable AEDs for individuals with ASD. Limited evidence supports the use of traditional non-AED treatments, such as the ketogenic and modified Atkins diet, multiple subpial transections, immunomodulation, and neurofeedback treatments. Although specific treatments may be more appropriate for specific genetic and metabolic syndromes associated with ASD and seizures, there are few studies which have documented the effectiveness of treatments for seizures for specific syndromes. Limited evidence supports l-carnitine, multivitamins, and *N*-acetyl-l-cysteine in mitochondrial disease and dysfunction, folinic acid in cerebral folate abnormalities and early treatment with vigabatrin in tuberous sclerosis complex. Finally, there is limited evidence for a number of novel treatments, particularly magnesium with pyridoxine, omega-3 fatty acids, the gluten-free casein-free diet, and low-frequency repetitive transcranial magnetic simulation. Zinc and l-carnosine are potential novel treatments supported by basic research but not clinical studies. This review demonstrates the wide variety of treatments used to treat seizures in individuals with ASD as well as the striking lack of clinical trials performed to support the use of these treatments. Additional studies concerning these treatments for controlling seizures in individuals with ASD are warranted.

## Introduction

A number of studies suggest that seizures affect a high proportion of children with autism spectrum disorder (ASD). Indeed, the reported prevalence of epilepsy in ASD ranges from 5 to 38%, which is clearly higher than the 1–2% prevalence in the general childhood population (1–5). A recent larger cross-sectional study suggests that the prevalence of epilepsy is 12.5% in children with ASD aged 2–17 years with this prevalence clearly highest for adolescents (6). In addition, the prevalence of treatment-resistant epilepsy in children with ASD is believed to be higher than in the general childhood population (7). Interestingly, recent reviews note shared cognitive symptoms in epilepsy and ASD, suggest a common etiopathophysiology (8), especially when ASD co-exists with intellectual disability (9). The overlap between ASD and epilepsy is also supported by studies that suggest treatment with anti-epileptic drugs (AEDs) in children with ASD can improve core and associated ASD symptoms.

Despite the fact that individuals with ASD and seizures appear to represent a large ASD subgroup, traditional seizure treatments for individuals with ASD have not been systematically reviewed and potential novel therapies have not been discussed. Although the first-line treatment for any child with seizures is AED therapy, the poor efficacy and/or adverse effects of AED treatments in individuals with ASD may prompt the use of traditional non-AED or novel treatments. Additionally, since seizures in ASD are associated with specific genetic and metabolic syndromes, therapies that target these syndromes may augment traditional treatments.

This review has three main purposes. First, in order to determine which traditional treatments for seizures are most effective and well tolerated in individuals with ASD, the evidence for the effectiveness of traditional seizure treatments is reviewed (see Traditional Treatments). Second, since seizures in ASD are associated with specific genetic and metabolic syndromes, therapies that target these syndromes are reviewed as they may augment traditional treatments (see Treatments for Specific Syndromes). Third, since there is a high prevalence of the use of novel treatments in ASD individuals with and without seizures (10) and in the general ASD population (11), novel treatments that have the potential to improve seizures are reviewed (see Novel Treatments). Overall, this review is designed to provide a comprehensive overview of the treatments for individuals with ASD who have comorbid seizures in order to achieve optimal outcomes.

## Methods

The Problem-Intervention-Comparison-Outcomes (PICOs) framework was used to conduct this review (12). The goal was to identify research studies which reported treatments that have the potential to improve clinical seizures for: (a) individuals with ASD and seizures, or (b) individuals with genetic and/or metabolic syndromes in which ASD and seizures are prominent features. We did not compare these treatments to other treatments and we considered all study designs. Our primary goal was to consider improvement in clinical seizure severity as the primary outcome. Since many treatments for children with ASD, especially seizure treatments, can have effects on core and associated ASD symptoms, as well as adverse effects (10), we also considered, as a secondary outcome, the effect of these treatments on core and associated ASD symptoms.

### Identification of potential treatments

Potential treatments for seizures in individuals with ASD were identified in several ways. First, we searched standard scientific databases. Second, we considered treatments associated with metabolic and genetic disorders that are commonly found in children with ASD and seizures. Third, we considered novel treatments obtained from two sources: (a) a panel of experts who regularly treat children with ASD and who attended at least one of the three Elias Tembenis Seizure Think Tanks (13), and (b) recent surveys of novel treatments used by parents in children with ASD and seizures and in the general ASD population (10, 11).

### Search strategy

A prospective protocol for this systematic review was developed *a priori*, and the search terms and selection criteria were chosen in an attempt to capture all pertinent publications. A computer-aided search of PUBMED, Google Scholar, CINAHL, EmBase, Scopus, and ERIC databases from inception through March 2013 was conducted to identify pertinent publications using the search terms “autism,” “autistic,” “Asperger,” “ASD,” “pervasive developmental disorder,” and “PDD” in combination with the terms “epilepsy,” ”epileptic,” and ”seizure.” The references cited in identified publications were also searched to locate additional studies. Three authors (Richard E. Frye, John C. Slattery, and Chrystal Lau) screened titles and abstracts of all potentially relevant publications. Studies were initially included if they: (a) involved individuals with ASD and epilepsy or seizures, and (b) reported at least one treatment for epilepsy or seizures. Articles were excluded if: (a) they involved animal models which did not specifically correspond to a well-known clinical syndrome; (b) were abstracts, posters, or conference proceedings that were not published in a journal; (c) did not present new or unique data (such as review articles or expert option); or (d) presented duplicate data.

### Level of evidence ratings

Although we considered conducting a meta-analysis on identified treatments, the lack of standard outcomes and the limitations in study design prevented a meta-analysis of any identified treatment. As an alternative, we provide a grade of recommendation (GOR) for each treatment based on the level of evidence. Using a well-established scale (14), each study was individually assessed to determine the level of evidence, ranging from Level 1 to 5 (see Table 1). After assessing all identified studies for each treatment, a GOR ranging from A (solid evidence) to D (limited, inconsistent, or inconclusive evidence) was assigned (see Table 2). Since a treatment could be a GOR of D for several reasons, we specified if the treatment received this rating because the evidence was a single case report or series (SC), was only based on bench research (BR), demonstrated a neutral effect (NE), or was found to be possibly detrimental (PD). If no studies were identified for a treatment, a GOR of N (no studies) was assigned.

**Table 1:** **Levels of evidence**.

Level	Description
1a	SR or meta-analysis of RCTs with homogeneity or Cochrane review with favorable findings
1b	Prospective high-quality RCT
2a	SR of cohort (prospective, non-randomized) studies with homogeneity
2b	Individual cohort (prospective, non-randomized) study or low-quality RCT
3a	SR of case-control (retrospective) studies with homogeneity
3b	Individual case-control (retrospective) study
4	Case series or reports
5	Expert opinion without critical appraisal or based on physiology or bench research

RCT, randomized controlled trial; SR, systematic review.

**Table 2:** **Grade of recommendation**.

Grade	Description
A	At least one Level 1a study *or* two Level 1b studies
B	At least one Level 1b, 2a, or 3a study, *or* two Level 2b or 3b studies
C	At least one Level 2b or 3b study, *or* two Level 4 studies
D	Level 5 evidence, *or* troublingly inconsistent or inconclusive studies of any level, *or* studies reporting no improvements
N	No studies identified

### Data analysis and synthesis

We summarized and synthesized the information about the various treatments in several ways. First, a GOR for: (a) treating behavioral and cognitive symptoms associated with ASD, (b) treating epilepsy in general, and (c) treating individuals with ASD and epilepsy, is provided in Table 3 for each treatment as well as important adverse effects. Also included in Table 3 is the percentage of parents reporting the use of specific treatments for children with and without seizures as reported in a recent survey study (10). A detailed discussion of the studies as well as the derivation of the ratings is discussed through Sections “Traditional Treatments,” “Treatments for Specific Syndromes,” and “Novel Treatments.” Second, using the available evidence, we provide recommendations for treatments for children with ASD and epilepsy (see Summary). Third, we discuss treatments that may have potential for future research study (see Summary). Fourth, a critique of the strengths and weakness of the studies on treatments for epilepsy in individuals with ASD is provided (see Summary).

**Table 3:** **Seizures treatments for autism spectrum disorder**.

Treatment	Grade of recommendation			Prevalence of use		Important adverse effects
	Seizures	Behavioral and cognitive ASD symptoms	ASD with seizures	Behavioral and cognitive ASD symptoms	ASD with seizures	
**TRADITIONAL TREATMENTS**						
Valproate	B	A	C	6%	39%	Weight gain (occasional), alopecia, carnitine depletion (infrequent); hepatotoxicity, abnormal liver function tests, hyperammonemia, and pancreatitis (rare)
Lamotrigine	C	D – NE	C	3%	27%	Stevens–Johnson reaction, abnormal liver function tests, aseptic meningitis (rare)
Levetiracetam	C	D – NE	C	1%	23%	Agitation and mood instability (infrequent)
Ethosuximide	B	D – NE	C	<1%	4%	Weight loss (occasional), gingival hyperplasia (infrequent)
Carbamazepine	B	D – SC	C	1%	26%	Dizziness, ataxia, nausea (occasional), hyponatremia, photosensitivity (rare)
Topiramate	B	D – PD	N	1%	25%	Weight loss, cognitive dysfunction, somnolence, agitation (occasional), metabolic acidosis, nephrolithiasis, glaucoma (rare)
Clonazepam	C	D – PD	N	3%	16%	Drowsiness, ataxia, cognitive dysfunction (occasional), abnormal liver function tests, respiratory depression (rare)
Oxcarbazepine	B	N	N	2%	22%	Dizziness, ataxia, nausea (occasional), hyponatremia, photosensitivity (rare)
Phenobarbital	B	D – PD	N	2%	13%	Drowsiness, lethargy, hyperactivity (common)
Phenytoin	B	D – PD	N	1%	14%	Dizziness, ataxia, nausea (occasional), abnormal liver function tests, gingival hyperplasia (infrequent)
Gabapentin	C	N	N	1%	8%	Weight gain, drowsiness, ataxia, cognitive dysfunction (occasional), edema (infrequent)
KD	B	B	C	0%	7%	Constipation, weight loss (occasional), nausea (infrequent), acidosis, nephrolithiasis (rare)
MAD	C	C	C	1%	3%	Constipation, slow weight gain (occasional), nausea (infrequent)
VNS	B	D – NE	D – NE	0%	4%	Hoarseness, throat discomfort (infrequent), shortness of breath, aspiration (rare)
Surgery – resection	C	D – PD	C	0%	<1%	Cognitive and motor dysfunction (occasional), intracranial hemorrhage (infrequent), intracranial infection (rare)
Multiple subpial transections	C	C	C			Cognitive and motor dysfunction, intracranial hemorrhage (infrequent), intracranial infection (rare)
Steroids	C	D – SC	D – SC	21%	5%	Hyperglycemia, hypertension, edema, poor wound healing, weakness, emotional dysregulation, weight gain, nausea, insomnia, gastrointestinal ulcer (occasional)
IVIG	C	C	N	1%	3%	Fever, nausea, rash (occasional), aseptic meningitis (rare)
Neurofeedback	B	B	C	<1%	<1%	None reported
**SPECIFIC SYNDROME AND TREATMENTS**						
Tuberous sclerosis complex						
Vigabatrin	B	C	C	0%	<1%	Retinal degeneration and central visual loss (infrequent)
Fragile X						
AEDs	C	N	N			Various (see above)
Mitochondrial disease						
l-carnitine/acetyl-l-carnitine	N	B	N	22%	18%	Abdominal discomfort, diarrhea, irritability (infrequent)
Multivitamins	N	B	N	16%	12%	Aggression, inattention, nausea (infrequent)
*N*-acetyl-l-cysteine	N	B	N			Abdominal discomfort, diarrhea, nausea (occasional)
Cerebral folate deficiency						
Folinic acid	C	C	C			Hyperactivity, aggression (rare)
Milk-free diet	N	C	N			Hypocalcemia (rare)
Urea cycle disorders						
Low-protein diet/ammonia binders/amino acid supplementation	B	C	N			None reported
Succinic semialdehyde dehydrogenase deficiency						
Vigabatrin	D – NE	D – NE	D – NE			Retinal degeneration and central visual loss (infrequent)
Creatine deficiency syndromes						
Creatine monohydrate	C	C	C			None reported
Biotinidase deficiency						
Biotin	D – NE	D – NE	D – NE			None reported
Smith–Lemli–Opitz syndrome						
Cholesterol	N	B	N			None reported
Branched-chain ketoacid dehydrogenase kinase deficiency						
BCAA supplement	D – PD	D – NE	D – NE			None reported
Pyridoxine-dependent and responsive seizures						
Pyridoxine	C	B	D – SC	27%	15%	Irritability, hyperactivity (uncommon)
Cobalamin metabolism						
Cobalamin	C	C	N	32%	20%	Irritability, hyperactivity (uncommon)
Organic aciduria – d-glyceric aciduria						
Fructose restriction	N	D – SC	D – SC			None reported
**NOVEL TREATMENTS**						
Magnesium (Mg)	A	D – NE	N	26%	17%	Abdominal discomfort, diarrhea, nausea (infrequent)
Pyridoxine and Mg	N	B	N			Abdominal discomfort, diarrhea, nausea, irritability, hyperactivity (infrequent)
Zinc	D – BR	N	N			Abdominal discomfort, diarrhea, nausea (infrequent)
Dimethylglycine	D – NE	D – NE	N	14%	9%	None reported
Taurine	D – PD	N	N	10%	10%	None reported
l-Carnosine	D – BR	C	N	5%	5%	Hyperactivity (infrequent)
Omega-3 fatty acids	C	B	N			Abdominal discomfort, diarrhea, nausea (infrequent), abnormal liver function tests (rare)
Homeopathy	N	D – PD	N			None reported
Gluten-free casein-free diet	N	B	C	41%	25%	None reported
Feingold/elimination diet	D – SC	N	N			None reported
Slow repetitive transcranial magnetic stimulation	B	B	N	<1%	<1%	Headache (occasional)

EO, Expert Opinion; NE, Neutral Effect / Inconsistent; PD, Possibly Detrimental; SC, Single Case Report or Series; BR, Based on bench research.

## Traditional Treatments

### Anti-epileptic drugs

#### Effectiveness of anti-epileptic drugs in autism spectrum disorder

There were no randomized control trials, cohort studies, or systematic reviews of case-control studies for any AED focusing on the control of seizure in the ASD population.

Two survey studies, one controlled and one uncontrolled, identified treatments used for seizures in individuals with ASD. The first study, conducted in the early 1990s, obtained the responses from 838 members of the Autism Society of North Carolina on a wide range of ASD treatments (15) (Level 4). Responders listed medications as well as their overall satisfaction with a class of drugs in general. Overall, 15.2% of the ASD individuals were receiving AED medications with the most common AEDs for the treatment of epilepsy being carbamazepine, valproic acid, and phenytoin, and the parents rated being satisfied with the AEDs in general.

**Figure 1:**
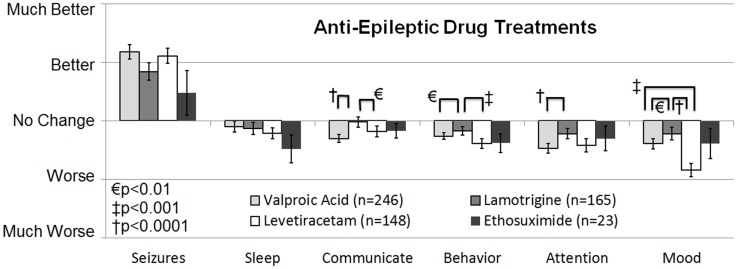
**Seizure survey rating for the most favorable rated anti-epileptic drug treatments for clinical seizures**. The perceived effect on seizures was not different across the four anti-epileptic drug treatments. Out of the four treatments lamotrigine appeared to worsen several other clinical factors, such as communication, attention, and mood less than valproate.

The second survey study, a retrospective national case-control survey study (Level 3b) conducted in 2010, determined whether a wide variety of treatments, including AEDs, were more beneficial than others treatments for individuals with ASD who also had seizures and/or epilepsy. Frye et al. (10) surveyed 733 parents of children with ASD who had seizures, epilepsy, and/or an abnormal electroencephalogram (EEG) and 290 parents of children with ASD but without these abnormalities as a control group. Parents rated the perceived effect of traditional AED and non-AED seizure treatments and non-traditional ASD treatments on clinical seizures and other clinical factors including sleep, communication, behavior, attention, and mood as well as three treatment side effects. A cluster analysis demonstrated that treatments could be broadly categorized into AED and non-AED treatments. For children with ASD and clinical seizures, AED treatments were found, on average, to improve seizures but worsened other clinical factors. When the AED treatments were specifically analyzed, four AEDs, specifically valproate, lamotrigine, levetiracetam, and ethosuximide, were found to provide the best seizure control and at the same time worsen other clinical factors the least out of all AEDs examined (see Figure 1). The other AEDs examined included phenytoin, clonazepam, carbamazepine, oxcarbazepine, topiramate, gabapentin, zonisamide, felbamate, and phenobarbital. All these other AEDs except phenobarbital were rated as significantly less beneficial for controlling seizures with a less favorable effect on other clinical factors, while phenobarbital was rated as having the most unfavorable effect on other clinical factors.

In a case series (Level 4) of 66 children with epilepsy, 50 with ASD, and 16 with ASD-like symptoms, performed in 1991, Gilbert reported a high response rate (with a low adverse effect rate) for valproate, carbamazepine, and ethosuximide (16). In another case series (Level 4) of 50 children, 28% with ASD, lamotrigine was found to have a favorable effect on seizures, particular in partial complex and absence seizures with a less favorable effect on tonic, tonic-clonic, and myoclonic seizures (17). Smaller case reports and case series (Level 4) have reported improvements in seizure frequency in children and adolescents with ASD using carbamazepine (18–20) and lamotrigine (17). Thus, there are many lower-quality (Level 4 and 3b) studies supporting the use of valproate, lamotrigine, levetiracetam, ethosuximide, and carbamazepine for the control of seizures in individuals with ASD, leading to a GOR of C for these AEDs. Other AEDs such as phenytoin, clonazepam, oxcarbazepine, topiramate, gabapentin, and phenobarbital, which are not uncommonly prescribed to children with ASD, do not have similar support in the literature and receive a GOR of N.

A recent systematic review of AED efficacy and effectiveness as initial monotherapy for epileptic seizures in children has been conducted by International League Against Epilepsy (21). Interestingly, many AEDs do not have well-controlled studies to support their use as monotherapy in children although stronger evidence is found for their use in adults with epilepsy. The ratings presented in Table 3 for these AEDs were abstracted from this systematic review. All AEDs except levetiracetam, ethosuximide, and gabapentin were abstracted from data on partial onset seizures in children. For levetiracetam, ethosuximide, and gabapentin there was no data on partial onset seizures in children so data from their efficacy for absence seizures in children was used.

#### Cognitive and behavioral effects of anti-epileptic drugs in autism spectrum disorder

Studies that have examined the behavioral and cognitive effects of AEDs in ASD are reviewed in this section.

##### Valproate

In randomized, prospective, double-blind, placebo-controlled (DBPC) studies (Level 1b), valproate monotherapy reduced repetitive behaviors (22) and irritability (23, 24) in individuals with ASD. In contrast, in an 8-week randomized, prospective, DBPC study (Level 1b) of aggressive ASD patients, improvements in irritability, aggression, or general clinical status were not different between the valproate treated and placebo groups (25), but many of the subjects maintained a sustained response to valproate in an open-label extension, and the participants who were weaned off valproate demonstrated relapse of symptoms. Thus, the researchers suggested that a large placebo response prevented them from ruling-out a true valproate effect. This study was complicated by the fact that the majority of the children had a significant intellectual impairment, children were excluded if they had a previous positive response to valproate, and children were tapered off all other psychotropic and anti-epileptic medication just prior to entering the trial.

Valproate has also been reported to improve behavioral and core ASD symptoms in a case series (Level 4) of ASD children with and without epilepsy (26) and to substantially improve ASD symptoms in case reports and series (Level 4) of children with subclinical epileptic-like discharges on EEG (27–32). In a case series (Level 4) of children with ASD or ASD-like symptoms and epilepsy, Gilbert reported that 41% treated with valproate demonstrated positive psychotropic effects (16). These studies provide good evidence that valproate can have beneficial cognitive and behavioral effects in individual with ASD. Given that at least two Level 1b studies have demonstrated positive effects and that these findings are supported by several additional studies, valproate is receives a GOR of A for the treatment of cognitive and behavioral symptoms in ASD.

##### Levetiracetam

In a prospective, open-label trial (Level 2b), levetiracetam improved attention, hyperactivity, emotional lability, and aggressive behaviors in six drug-naïve boys but not in four boys who had been recently weaned off psychotropic medications (33). However, no significant improvement or worsening of aberrant or repetitive behaviors or impulsivity or hyperactivity was found in a small prospective, randomized, DBPC trial (Level 2b) of levetiracetam (34). Thus, there is insufficient evidence to suggest that levetiracetam improves behavioral or cognitive features of ASD; however, these studies suggest that levetiracetam is well tolerated without detrimental cognitive or behavioral effects. Thus, levetiracetam is given a GOR of D – NE for neutral effect on ASD cognitive and behavioral symptoms.

##### Lamotrigine

Lamotrigine did not improve or worsen aberrant or ASD behaviors in a small prospective, randomized, DBPC study (Level 2b) (35) but in a case series (Level 4) of 50 children, 28% with ASD, parents reported improvements with lamotrigine in cognitive and ASD symptoms in 62% of children with ASD who had intractable epilepsy, even if seizure frequency did not improve (17). Although lamotrigine may be cognitively enhancing in non-ASD epileptic individuals (36) and is efficacious for mood stabilization in bipolar disorder (37), there is insufficient evidence to suggests that it improves behavioral or cognitive features of ASD; rather these studies suggest that lamotrigine has few detrimental cognitive or behavioral effects in individuals with ASD. Thus, lamotrigine is given a GOR of D – NE for neutral effect on cognitive and behavioral symptoms in individuals with ASD.

##### Topiramate

In individuals with ASD, topiramate, when added on to risperidone, reduced irritability, stereotypical behavior, and hyperactivity in a DBPC study (Level 2b) (38) but caused behavioral adverse effects in some participants in an open-label study (Level 4) (39). Given the inconsistent results and the fact that topiramate can have neurocognitive adverse effects in individuals with epilepsy (40), topiramate is probably only helpful for the treatment of behavior in selected individuals with ASD. Thus, topiramate is given a GOR of D – PD for possible detrimental effect on cognitive and behavioral symptoms in children with ASD.

##### Other AEDs

In a case series (Level 4) of children with ASD or ASD-like symptoms and epilepsy, Gilbert reported that 56% treated with carbamazepine demonstrated positive psychotropic effects (16). Thus, carbamazepine receives a GOR of D – SC for because the evidence is limited to a single case-series. In a retrospective case-control survey study (Level 3b) ethosuximide was rated by parents to be one of four AEDs that had the least detrimental effect on behavioral and cognition but there was no ratings of improvements in cognitive or behavioral symptoms (10). Thus, ethosuximide receives a GOR of D – NE for a neutral effect on behavioral and cognition in ASD.

In a case series (Level 4) of 66 children with epilepsy, 50 with ASD, and 16 with ASD-like symptoms, Gilbert reported a high prevalence of extremely negative behavioral adverse effects for clonazepam, phenytoin, phenobarbital, and nitrazepam (16). Given that phenytoin, clonazepam, and phenobarbital were in the group of AEDs rated as having detrimental behavioral and cognitive effects on children with ASD in a retrospective case-control survey study (Level 3b) (10), these AEDs receive a GOR of D – PD for possible being detrimental for behavioral and cognitive symptoms of ASD.

### Traditional non-anti-epileptic drug treatments

#### Ketogenic and modified Atkin’s diet

**Figure 2:**
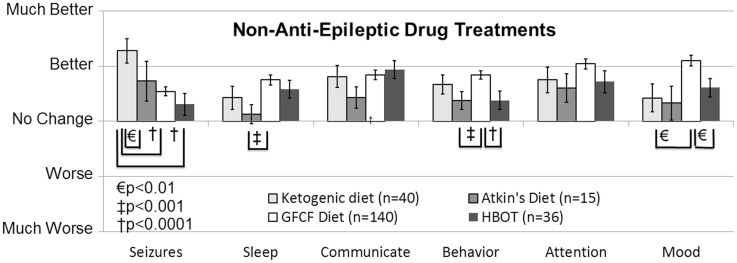
**Seizure survey ratings for the most favorably rated non-anti-epileptic drug treatments for clinical seizures**. The ketogenic diet was perceived to improve seizures more than the gluten-free-casein-free diet and hyperbaric oxygen treatment. Other clinical factors were not perceived to differ significantly between these four treatments except for behavior, sleep and mood, which was significantly better for the gluten-free casein-free diet as compared to some other treatments.

The ketogenic diet (KD) has a long and successful history for treating epilepsy, especially for AED refractory epilepsy, and some researchers have suggested it as a first-line therapy. Several unblinded but randomized controlled trials (Level 2b) conducted in children with seizures have reported an effect comparable to AEDs (41), resulting in a GOR of B for the treatment of seizures. Since long-term compliance with the KD can be difficult because of the very high levels of fat (90% of calories), the modified Atkins diet (MAD) is sometimes used since it may have better long-term patient compliance. Studies on children and adults with epilepsy suggest similar efficacy with the KD and MAD (Level 2b) (42), resulting in a GOR of C for the MAD for seizure control. In a retrospective case-control survey study (Level 3b), the KD was rated as being the most favorable non-AED treatment for improving seizures, and also was rated as providing favorable effects on other important clinical factors related to ASD (Figure 2) (10). In addition, a small prospective, uncontrolled study demonstrated a favorable response rate for the improvement of ASD symptoms (Level 2b) (43). Another recent case report (Level 4) demonstrated that the KD significantly improved seizures in a 12-year-old girl with ASD (44). Thus the KD receives a GOR of B for treatment of ASD cognitive and behavior symptoms and GOR of C for treatment of epilepsy in individuals with ASD. The MAD also received favorable ratings in the retrospective case-control survey study (Level 3b) for the control of seizures and other behavioral and cognitive ASD symptoms, although not as favorable as the KD (10). Thus, the MAD receives a GOR of C for the treatment of behavioral and cognitive ASD symptoms and for controlling seizures in individuals with ASD. Overall these are promising interventions for the treatment of seizures in individuals with ASD, especially in those with drug-resistant seizures. More research is clearly warranted.

#### Vagus nerve stimulator

In adults and adolescents with drug-resistant epilepsy, the vagal nerve stimulator (VNS) has been shown to reduce seizure frequency in randomized controlled studies (Level 2b) (45), resulting in a GOR of B for seizure control. A retrospective review of 59 ASD patients with seizures who were treated with VNS found that more than half experienced at least a 50% reduction in seizure frequency, and more than half demonstrated significant quality of life improvements (Level 4) (46). The VNS was reported to improve seizures and behavior in an adult with Asperger syndrome and bitemporal epilepsy (Level 4) (47). However, a prospective trial of eight children and adolescents with medically intractable epilepsy and ASD found no improvement in seizure frequency or cognition, and less than half of the cases demonstrated minor improvements in general functioning (Level 2b) (48). Lastly, in a case series, only two of nine patients with treatment-resistant epilepsy and ASD demonstrated limited improvement with the VNS (Level 4) (49). Given these conflicting results, the VNS receives a GOR of D – NE for neutral effect for the treatment of seizures and cognitive and behavior symptoms in individuals with ASD. Given the fact that the VNS requires operative placement which could lead to complications, it is probably wise to carefully consider VNS treatment in children with ASD and treatment-resistant epilepsy after other epilepsy treatments have failed.

#### Surgery

In general, the most efficacious routine epilepsy surgery involves resection of a cortical area in which epileptic foci clearly arises. Straightforward situations occur where potential epileptic foci are clearly visualized on magnetic resonance imaging, such as Tuberous Sclerosis Complex (TSC) and tumors, particularly temporal lobe tumors (50–52). However, even when a focus is visualized, it is essential to verify the epileptic focus. For individuals without magnetic resonance imaging-confirmed foci, procedures for localizing epileptic foci, such as magnetoencephalography (MEG), subdural intracranial recording grids, and/or intraoperative mapping, are necessary. Given the difficulty with performing blinded or case-control studies in a surgical population, systematic reviews have examined many case series (Level 4) of outcomes from lesional surgery (53). Thus, epilepsy surgery in general is given a GOR of C because of multiple Level 4 studies documenting effectiveness.

Several case series have examined whether ASD symptoms and cognition change after epilepsy surgery in children with ASD and treatment-resistant epilepsy. Of five children with resection of focal lesions, improvement could only be confirmed by neuropsychological testing in one case and another child clearly became worse (Level 4) (54). In a case series of two boys with focal epilepsy and TSC-like lesions, only one child was clearly improved but only years after the surgery (Level 4) (55). In a series of 24 epilepsy surgery cases, none of the 7 children with ASD had a change in their ASD diagnosis or demonstrated an improvement in intelligence (Level 4) (56). In another case series of 60 children, 38% who had ASD, temporal lobe resection to control seizures did not result in an overall change in psychiatric diagnosis (Level 4) (57). However, in another case series of 16 patients, 5 who had ASD, 3 of these ASD patients demonstrated a positive behavioral change, whereas 1 ASD patient demonstrated no change, and another 1 worsened (Level 4) (58). Thus, there are several Level 4 studies demonstrating that standard epilepsy surgery (i.e., resection) does not consistently improve ASD symptoms or cognition and can result in worsening of behavioral and cognitive outcomes, thus such surgery receives a GOR of D – PD for being potentially detrimental.

In some children with autistic epileptiform regression (AER), multiple subpial transections (MSTs) have been studied (59–61) but this type of surgery is not common. Favorable results have been reported in two children with AER after MST (Level 4) (59) but a slightly larger case-series suggests that any improvement is limited and temporary (Level 4) (61). Impressive results from MST were reported in a case series (Level 4) of 18 children with ASD who underwent extensive extra- and intra-operative electrocorticography pre-surgical workups, including MEG, to guide subdural grid placement (Level 4) (60). Most of these cases were found to have many independent foci, required MST in several different cortical areas, and all children were also treated with steroids. In this series, 4, 8, and 6 children demonstrated major, moderate, and minor improvements, respectively. None of the children demonstrated worsening. This latter study suggests that optimal outcomes can be obtained when MST is applied to multiple foci in combination with steroids. MST receives a GOR of C for improving seizures and behavioral symptoms in children with ASD, particularly the subgroup with AER, since several case-series support this notion. MST has been documented to be effective in seizure reduction in children with intractable epilepsy without ASD in several case series (Level 4) (62–64), resulting in a GOR of C.

#### Immunomodulatory treatments

Immunomodulatory treatments, particularly steroids and intravenous immunoglobulin (IVIG), have been used to treat many drug-resistant epilepsy syndromes as well as those which are believed to have an autoimmune basis. Such treatments are particularly useful in Landau–Kleffner syndrome and Continuous Spike-Wave Activity during Slow-Wave Sleep, both of which are syndromes that share some characteristics with ASD.

Despite the relatively common use of steroids in the treatment of ASD (see Table 3), only two case reports (Level 4) of children with normal EEGs and non-epileptic autistic regression documented improvements in language and behavior following corticosteroid treatment (65, 66). Steroid treatment has been documented to have positive effects on improvements in seizures and EEG activity in children with ASD when used in combination with MST in a case series of children with ASD and subclinical epileptiform discharges (Level 4) (60) and in combination with valproic acid in an open-label prospective study (Level 2b) (67). Thus, for the treatment of ASD behaviors and epilepsy in individuals with ASD, steroid treatment has a GOR of D – SC because of the limited reports documenting its usefulness. Steroids have been documented to be effective in seizure reduction in children with intractable epilepsy without ASD in several case series (Level 4) (68, 69), resulting in a GOR of C.

A relatively large case series (Level 4) (70) and a case series (Level 4) using a standardized treatment protocol (71) have documented the benefit of IVIG in children with ASD without epilepsy and one of these studies demonstrated regression with discontinuation of IVIG treatment (70). In contrast, other smaller case series (Level 4) (72) and case series (Level 4) using non-standardized protocols (73) were not able to confirm such improvements (overall GOR of C for the treatment of ASD symptoms). Although IVIG is not uncommonly used in drug-resistant epilepsy, the evidence for its efficacy remains documented in case series (Level 4) (74–77) (overall GOR of C for the treatment of seizures in general). IVIG treatment receives a GOR of N for seizures in ASD since there are no reports for its use in this context.

#### Neurofeedback

Neurofeedback is a safe treatment that uses operant conditioning to increase coherence between seizure prone and non-seizure prone brain regions. Coherence information is integrated into an auditory, visual, or audiovisual game. The patient succeeds at the game by increasing coherence between selected brain regions (78). Two recent meta-analyses (Level 3a) suggest that neurofeedback can reduce seizures in a majority of patients, even in patients with otherwise uncontrolled epilepsy (79, 80), supporting a GOR of B. Research suggests that neurofeedback may be an effective form of cognitive therapy for children with ASD (78) and a recent review has outlined seven controlled studies (81) with at least three prospective studies using a non-blind wait-list control group (82–84) (Level 2b). Thus, the use of neurofeedback for behavioral and cognitive symptoms of ASD receives a GOR of B for treating ASD symptoms. Several case studies suggest that it may be helpful for seizure disorders in ASD (Level 4) (85–87), supporting a GOR of C.

## Treatments for Specific Syndromes

Autism spectrum disorder is associated with several metabolic and genetic syndromes in which at least some of the pathophysiology is known. Some of these syndromes have treatments which could improve the underlying pathophysiological processes and, thus, the associated seizures. In this section, we review genetic and metabolic disorders associated with ASD and seizures and discuss potential treatments.

### Tuberous sclerosis complex

In one case series, vigabatrin, a γ-aminobutyric acid (GABA) transaminase inhibitor that increases GABA concentrations in the brain, was found to be particularly effective for infantile spasms and partial seizures in TSC patients with ASD (Level 4) (88). Vigabatrin also improved core ASD symptoms in some patients in this case series (Level 4) (88). In two case series (Level 4), starting vigabatrin soon after the onset of seizures lowered the risk of developing ASD symptoms as compared to a delay in starting vigabatrin treatment (Level 3b) (89, 90). In one case report (Level 4), a child with TSC demonstrated AER after vigabatrin treatment was discontinued, suggesting that continued treatment with vigabatrin may have prevented the development of ASD (91). Since several case series and reports have documented the potential beneficial effect of vigabatrin for seizures, particularly infantile spasms, and cognitive and behavioral symptoms in ASD, this treatment receives GOR of C for both ASD symptoms and seizures, particularly when started early in life. A small prospective, randomized, multicenter controlled trial (Type 1b) has demonstrated the effectiveness of vigabatrin for infantile spasms in TSC (92), resulting in a GOR of B for seizure control in TSC.

### Fragile X

Fragile X, the most commonly inherited autism syndrome, accounts for approximately 1–5% of ASD cases (93). An imbalance between the excitatory metabotropic glutamate receptor and the inhibitory GABA receptor is believed to cause many of the behavioral, cognitive, and epileptiform symptoms in Fragile X syndrome. Although seizures appear to frequently occur (10–20% prevalence) in Fragile X, case series, and survey studies (Level 4) suggest that they are usually easily treatable with standard AED therapy (94–97). Mouse models (Level 5) have demonstrated that novel glutamate receptor modulators, such as the inverse agonist 2-methyl-6-(phenylethynyl) pyridine (98), as well as GABA agonists, such as baclofen and ganaxolone (99), can increase seizure threshold. Similar approaches are being used in clinical trials to treat ASD symptoms in Fragile X patients (100). In addition, mouse models (Level 5) have demonstrated that other novel treatments such as lovastatin can reduce excess hippocampal protein synthesis leading to reduced epileptogenesis (101) and that addressing a central metabolic regulatory abnormality with glycogen synthase kinase-3 inhibitors reduces seizures (102).

### Mitochondrial disease and dysfunction

Mitochondrial dysfunction appears to be one of the most prevalent metabolic disorders in ASD (103). One of the first descriptions of a mitochondrial disease (MD) associated with ASD was the HEADD syndrome, an association of hypotonia, epilepsy, autism, and developmental delay, which was described in a series of ASD children with respiratory chain disorders (104). Seizures are present in an estimated 41% of children with ASD and MD (103).

No treatments have been systematically studied for the treatment of seizures in children who have MD with or without ASD (105, 106). Children with ASD and MD have been treated with a variety of MD treatments including carnitine, co-enzyme Q10, B vitamins, and vitamins C and E (107). Certain standard treatments for MD have been shown to improve core and associated symptoms of ASD in controlled studies. Interestingly, these treatments have not undergone rigorous study for the treatment for MD and are used largely on the basis of expert opinion. Treatments used for MD that have been studied in ASD include l-carnitine in two DBPC studies (Level 2b) (108, 109) resulting in a GOR of B for ASD symptoms; multivitamins containing B vitamins, antioxidants, vitamin E, and co-enzyme Q10 in two DBPC studies (Level 2b) (110, 111) resulting in a GOR of B for ASD symptoms; and *N*-acetyl-l-cysteine in two DBPC trials (Level 2b) (112, 113) and a case report (Level 4) (114) resulting in a GOR of B. Although the efficacy of improving seizures with standard treatments for MD is not known, resulting in a GOR of N for seizures, it is logical to assume that improving mitochondrial function may reduce seizures.

### Abnormalities in folate metabolism

Folate is essential for a wide range of important metabolic processes, including oxidative pathways, homocysteine metabolism, and gene methylation (115). Disruption in any of these processes could result in an increased susceptibility to developing seizures. ASD has been associated with several polymorphisms that can decrease production of 5-methyltetrahydrofolate (5MTHF) and impair folate transport across the blood-brain barrier and into neurons, including polymorphisms in methylenetetrahydrofolate reductase (116–125), dihydrofolate reductase (126), and the reduced folate carrier (117). More significant is impairment of the transport of 5MTHF across the blood-brain barrier by dysfunction of the folate-receptor alpha (FRα) (127). This transport system can be blocked by the FRα autoantibody which may be present in a majority of children with ASD (127, 128) or can be dysfunctional in ASD children with MD (129). Individuals with the FRα autoantibody are typically treated with 0.5–2 mg/kg/day of folinic acid, while individuals with MD may require 4+ mg/kg/day of folinic acid. In addition, a milk-free diet has also been shown to reduce serum FRα autoantibody titers in a controlled study (130). Multiple small and large case series (Level 4) have demonstrated that folinic-acid treatment in ASD children with FRα autoantibody can result in partial improvements in communication, social interaction, attention, and stereotypical behavior (127, 130–133) to complete recovery of ASD symptoms (132, 134), resulting in a GOR of C for ASD symptoms. Two case series (Level 4) have noted improvement in seizures in children with ASD and cerebral folate abnormalities with folinic acid treatment resulting in a GOR of C for ASD and seizures (133, 134). In several case reports and case series (Level 4), folinic acid has been shown to treat refractory epilepsy in a disorder called folinic-acid responsive seizures (135, 136), giving it a GOR of C for epilepsy. Interestingly, the folinic-acid responsive seizure syndrome is now understood to overlap with pyridoxine-responsive seizures (137). The milk-free diet has been shown to decrease the serum concentration of the FRα autoantibody and improve irritability in ASD in a controlled study (Level 2b), resulting in a GOR of C for ASD symptoms. The milk-free diet has not been studied for seizures in children with or without ASD (GOR of N).

### Urea cycle disorders

Two cases of ASD children with urea cycle disorders, one with ornithine transcarbamylase deficiency and arginase deficiency (138) and another with carbamoyl phosphate synthetase deficiency (139) have been reported to date. Standard therapy is focused on reducing ammonia through a low-protein diet and ammonia binders as well as supplementation with specific amino acids and various vitamin supplements (140). Improvement in ASD symptoms has been reported with treatment in these case reports (Level 4), resulting in a GOR of C. Neither one of these cases had seizures, resulting in a GOR of N for ASD and seizures. Seizures and neurological symptoms have been shown to improve with standard therapy in individuals with urea cycle disorder in large cohort (Level 2b) and case series (Level 4) studies resulting in a GOR of B (141).

### Succinic semialdehyde dehydrogenase deficiency

Succinic semialdehyde dehydrogenase deficiency is a rare autosomal recessive disorder of GABA metabolism that includes ASD features and seizures (142). In the absence of succinic semialdehyde dehydrogenase, GABA is degraded by an alternative pathway that produces gamma-hydroxybutyric acid. Vigabatrin inhibits the formation of succinic semialdehyde but has been inconsistent in the control of seizures and cognitive improvement, resulting in a GOR of D – NE for inconsistent result (142).

### Creatine deficiency syndromes

Creatine and phosphocreatine play important roles in energy storage and transmission of high-energy phosphates. Three inborn disorders of creatine metabolism, collectively known as the creatine deficiency syndromes, include symptoms of developmental delay, regression, ASD features, mental retardation, language disorders, dyskinesia, and seizures (143). Several case series (Level 4) and case reports (Level 4) have documented that creatine deficiency disorders can be treated with high doses of creatine monohydrate along with restriction and supplementation of specific amino acids (GOR of C) (141).

### Biotinidase deficiency

Disorders of biotin (vitamin B7) metabolism manifest with seizures and developmental delays. One child with ASD has been reported to have a partial biotinidase deficiency but did not respond to biotin supplementation (Level 4). However, the younger brother who also manifested symptoms of partial biotinidase deficiency did not develop ASD potentially due to treatments started early in life (Level 4) (144). Biotin supplementation has not been documented to improve seizures in biotinidase deficiency (141). Thus, biotin receives a GOR of D – NE for a neutral effect on seizures, ASD symptoms, and seizures in ASD.

### Smith–Lemli–Opitz syndrome

Many (50–75%) children with Smith–Lemli–Opitz syndrome meet criteria for ASD (145, 146). Treatment with cholesterol supplementation in children with Smith–Lemli–Opitz syndrome has been reported to improve ASD and associated behavioral symptoms in a case report (147) (Level 4), case series (148) (Level 4), and prospective cohorts (149, 150) (Level 2a), especially in young children (151), although a short DBPC trial (Level 2b) in mostly children and adolescents did not confirm such findings (152). Thus, cholesterol supplementation receives a GOR of B for young children with Smith–Lemli–Opitz syndrome for behavioral ASD symptoms. Cholesterol supplementation has not been studied for seizure control for Smith–Lemli–Opitz syndrome in children with or without ASD resulting in a GOR of N.

### Branched-chain ketoacid dehydrogenase kinase deficiency

Inactivating mutations in the branched-chain ketoacid dehydrogenase kinase gene were identified in two consanguineous families with ASD, epilepsy, and intellectual disability. This mutation resulted in reduced levels of branched-chain amino acids. Treatment with a diet enriched in branched-chain amino acids resulted in reversal of neurological abnormalities in the mouse model (Level 5) and normalization of amino acids without clinical improvement in a case series of patients (Level 4) with this mutation (153). Interestingly, one study of genetic absence epilepsy rats from Strasbourg, a genetic model of generalized non-convulsive epilepsy (Level 5), reported increased seizures with an intraperitoneally injection of branched-chain amino acids, presumably due to the role of these amino acids on glutamate metabolism (154). Thus, dietary enrichment of amino acids probably should not be used in epilepsy in general because of the possible detrimental effects (GOR of D – PD for possible detrimental), and only be carefully considered in the context of known branched-chain ketoacid dehydrogenase kinase deficiency as the evidence for improvement on ASD symptoms and/or epilepsy is lacking and not consistent with the animal model (GOR of D – NE for inconsistency).

### Pyridoxine-dependent and pyridoxine-responsive seizures

Pyridoxine is a cofactor for over 110 enzymes. Pyridoxine is effective for treating children with pyridoxine-dependent or pyridoxine-responsive seizures (155–158). The effectiveness of pyridoxine in pyridoxine-dependent and pyridoxine-responsive seizures has been documented in many case studies (Level 4) (159), resulting in a GOR of C. When pyridoxine-dependent and pyridoxine-responsive seizures are suspected, it is important to remember that pyridox(am)ine phosphate oxidase deficiency and neonatal/infantile hypophosphatasia are in the differential diagnosis and that co-treatment with folinic acid and a lysine restricted diet should also be considered (159).

The notion of abnormal pyridoxine metabolism was first suggested when a subset of children with ASD were found to have abnormalities associated with pyridoxine-dependent enzymes on a tryptophan loading test (160). Several open-label trials demonstrated positive results for vitamin B6 (161, 162). For example, in a large open-label (Level 2b) study, children with ASD were shown to have global improvement with a vitamin combination that included high dose (150–450 mg) pyridoxine (162). A small randomized DBPC cross-over variable-dose (75–3000 mg) withdrawal study (Level 2b) demonstrated that significantly more children with ASD demonstrated improvement on pyridoxine (73%) as compared to the placebo (20%) (163). A DBPC cross-over trial (Level 2b) which evaluated pyridoxine as well as several other treatments for ASD symptoms did not demonstrate efficacy of pyridoxine as compared to baseline (164). However, this latter study may have been flawed since it did not compare the only pyridoxine group to placebo. Thus, pyridoxine receives a GOR of B for treatment of behavioral and cognitive ASD symptoms. However, there is larger support for the use of pyridoxine in combination with magnesium to treat ASD symptoms (see section below).

To date, there has been one case report of ASD associated with severe mental retardation, aerophagia, breath holding, self-injury and pyridoxine-dependent seizures (165). According to this report, high-dose pyridoxine improved seizures but it was not possible to measure whether it improved ASD characteristics (Level 4), therefore pyridoxine receives a GOR of D – SC for treatment of seizures in individuals with ASD.

### Abnormalities in cobalamin metabolism

Cobalamin deficiency is uncommonly associated with seizures (166, 167), with the most common presentation including megaloblastic anemia, feeding difficulties, developmental delay, microcephaly, failure to thrive, hypotonia, lethargy, irritability, involuntary movements, and cerebral atrophy. Seizures may be focal, multifocal, and/or myoclonic (166, 167), may develop after treatment starts (166) and have been documented to improve with cobalamin supplementation in many case reports (Level 4) (166, 167). Thus, cobalamin supplementation for seizures in the context of cobalamin deficiency receives a GOR of C.

Children with ASD have been shown to have abnormalities in cobalamin (vitamin B12) dependent metabolism (117). A recent prospective open-label study (Level 2b) demonstrated that glutathione, but not methylation, metabolism could be improved in children with ASD following a 3-month supplementation with methylcobalamin injection and oral folinic acid (168). This study did not report the effects of the therapy on ASD cognition or behavior, but a small, prospective, open-label study (Level 2b) demonstrated significant improvements in core ASD symptoms as well as intelligence and developmental quotient with methylcobalamin injections (169). Thus, cobalamin receives a GOR of C for treatment of behavioral and cognitive abnormalities in ASD and a GOR of N for seizures in individuals with ASD since there are no supporting studies.

### Organic acidurias

Although several organic acidurias have been reportedly associated with ASD in case studies, including d-glyceric aciduria (170), propionic acidemia (171), and l-2-hydroxyglutaric aciduria (172), only d-glyceric aciduria has been associated with seizures (170). In this latter case report, seizures and ASD symptoms improved with fructose restriction (170). Since the use of fructose restriction has only been reported in this one case, this therapy receives a GOR of D – SC for treatment of ASD symptoms and seizures and a GOR of N for epilepsy as there are no studies supporting such a treatment. In addition, since such therapy is specific to a particular organic aciduria, it is unlikely to be a candidate as a novel therapy for the treatment of epilepsy in ASD or other metabolic disorders.

## Novel Treatments

Considering the wide use of novel treatments in the ASD population (10, 11, 173), a discussion of such treatments as adjunctive therapy in seizure control deserves consideration. Here we review some of the more commonly used novel therapies in ASD that have some evidence for treating seizures.

### Magnesium (Mg)

Ionized Mg (Mg^2+^) has antagonistic effects on the *N*-methyl-d-aspartate receptor (174, 175) and magnesium deficiency may be a factor in several forms of epilepsy (176–178). It has been suggested that low Mg^2+^ or altered balance between Mg^2+^ and ionized calcium (Ca^2+^) may precipitate seizures (179). Patients with epilepsy have been shown to have significantly lower mean Mg^2+^ levels and an increased Ca^2+^/Mg^2+^ ratio in spite of normal total serum Mg levels (180). Mg has been shown to decrease the seizure duration in idiopathic epilepsy with the reduction in duration proportional to the severity of the seizure (181).

A review of controlled trials (Level 1a) of Mg sulfate (MgSO_4_) use for the prophylaxis and management of eclamptic seizures demonstrated its overwhelming evidence as the standard of care (182). In a small, open-label study (Level 2b) on infantile spasms, adding MgSO_4_ to ACTH resulted in improvement in the resolution of seizures (183). A small, retrospective, chart review (Level 4) suggested that adjunctive oral Mg treatment resulted in substantial improvement in seizure frequency in patients with refractory epilepsy (184). Based on case series, Mg has been recommended as second line therapy in status epilepticus in an expert review (Level 5) (185) and has been reported to be therapeutic in the treatment of refractory status epilepticus in two girls with juvenile-onset Alpers’ syndrome (Level 4) (186). The significant evidence for Mg as a treatment for seizures results in a GOR of A.

In children with ASD, Mg in combination with pyridoxine is used as a novel treatment, and Mg alone is used for the treatment of constipation but has undergone few clinical studies. In one DBPC cross-over study (Level 2b), a comparison between Mg and placebo demonstrated mixed results in treatment of ASD symptoms (164). Given the mixed results, Mg receives a GOR of D – NE for inconsistent results for the treatment of ASD symptoms. Mg supplementation has not been investigated in children with ASD who have epilepsy resulting in a GOR of N. However given that it is helpful in other conditions with seizures, it has the potential to help children with ASD who also have seizures.

### Combined pyridoxine and magnesium therapy

An early study suggested positive behavioral effects of combined pyridoxine and Mg in children with ASD in a moderately sized, open-label, study (Level 2b) (187). In a hybrid designed study, about 34% of 44 ASD children were found to be responders in a initial open-label phase study with their response verified in an subsequent DPBC phase (Level 2b) (188). In a rather complicated medium-size DBPC cross-over design (Level 2b), the combination of pyridoxine and Mg was shown to be superior to each treatment alone and to placebo (164). However, more recent small DBPC studies (Level 2b) have not identified an effect of either high-dose (189) or low-dose (190) combination therapy for children with ASD. A recent uncontrolled (Level 4) study demonstrated that the combination of pyridoxine, Mg, and riboflavin significantly reduced the levels of urine dicarboxylic acid in children with ASD (191). Combination pyridoxine and Mg therapy has not been used in epilepsy in children with or without ASD, although each component alone has been used (see above). Thus, for epilepsy with or without ASD, this combination receives a GOR of N. A recent review of novel therapies for use in ASD did suggest that this combination therapy was acceptable with careful monitoring (173) and given the studies reviewed above, the combination receives a GOR of B for cognitive and behavioral symptoms in ASD. Given that these therapies might have benefit effects in selected children with ASD and epilepsy, the combination therapy may be beneficial and should undergo further research.

### Zinc

Two recent studies have demonstrated that plasma zinc (Zn) is reduced in children with ASD (192, 193), while two other studies have not supported such findings (194, 195). Zn is essential for immune, hormone, antioxidant, genetic, neurological, and physiological processes. Low Zn has been found in the serum and cerebrospinal fluid of children with febrile seizures in case-control studies (196, 197) and was associated with adult epilepsy in one case-control study of 40 patients and matched healthy controls (198) and also with idiopathic intractable epilepsy in one case-control study of 70 participants matched on age, ethnicity, and socioeconomic status (199). Although Zn has been shown to decrease the duration of excitatory response of dentate granule cells derived from patients with medial temporal lobe epilepsy (Level 5) (200) and decrease seizure susceptibility in the EL mouse model (Level 5) (201), it has not undergone any clinical trials as a treatment for epilepsy. Thus, it receives a GOR of D – BR for treatment for seizures because the evidence is based on only bench research. Although there is no evidence to support Zn supplementation in the treatment of epilepsy in ASD or for improving behavior and cognition in ASD (GOR of N), given the important role of Zn in neuronal function, the supportive laboratory data, and the fact that children with ASD might already have a deficiency, Zn supplementation may be an important novel treatment to investigate in the future.

### Dimethylglycine

There have been mixed reports of effectiveness of dimethylglycine (DMG) on seizures. In a single-subject repeated-measures design (Level 4), a significant reduction in seizures in a 22-year-old male with mental retardation and intractable epilepsy was reported with the use of 90 mg of DMG twice daily (202). However, small clinical trials have not verified the efficacy of DMG. No reduction in seizure frequency was found in five participants with refractory seizures given 270 mg of DMG/day over a 1-month period in a DBPC cross-over study (Level 2b) (203). No reduction in seizure frequency was found between the control and treatment groups in a DBPC study of 20 patients with mental retardation and seizures treated with 300 mg of DMG/day during the first 2 weeks of the study, and then 600 mg/day during the second 2 weeks of the study (Level 2b) (204). DMG receives a GOR of D – NE for inconsistent evidence for the treatment of seizures. DMG has been evaluated in two small DBPC (Level 2b) for children with ASD, both of which demonstrated no significant benefit for ASD symptoms (205, 206). DMG receives a GOR of D – NE for inconsistent evidence of effectively treating ASD symptoms. DMG has not been studied in a controlled fashion in children with ASD and seizures so it receives a GOR of N. This evidence does not support further investigation of DMG as a novel treatment in individuals with ASD and seizures.

### Taurine

Taurine is a sulfur containing organic acid that may have neuroprotective and neuromodulating properties. Taurine has been proposed to be a weak GABA_A_ and GABA_B_ receptor antagonist with potential anticonvulsant activity (207–211) but its ability to cross the blood-brain barrier is limited (212) and studies have not been conducted to determine if taurine could be an efficacious therapy for epilepsy (213). Two studies found that plasma taurine levels were significantly lower in children with ASD compared to controls (195, 214) while another study reported that taurine improved learning in Fragile X mice (215). Taurine has been studied in two open-label treatment studies of children and adults with drug-resistant epilepsy without a noted sustained beneficial effect (Level 2b) (216, 217) while two open-label studies of taltrimide, a taurine derivative, demonstrated no clinical effect in one study and a proconvulsive effect in the other study (Level 2b) (218, 219). Thus, taurine receives a GOR of D – PD for having possible detrimental effects for the treatment of seizures. The effect of taurine on children with ASD and epilepsy has not been systematically studied resulting in a GOR of N. Given the fact that it has not been found to be effective in children with epilepsy, there is little support for its use in children with ASD and epilepsy.

### Carnosine

Carnosine (*B*-alanyl-l-histidine), a naturally occurring dipeptide, not only serves as a source of histidine, a precursor of histamine (220, 221), but unlike histamine, it is able to cross the blood-brain barrier. l-Carnosine has been demonstrated to significantly improve the Gilliam Autism Rating Scale and the Receptive One-Word Picture Vocabulary tests without any adverse effects in an 8-week DBPC study in children with ASD (Level 2b) (222). With only one Level 2b study, l-carnosine receives a GOR of C for the treatment of cognitive and behavioral symptoms of ASD.

Histamine’s involvement in mechanisms regulating seizure susceptibility has been documented in multiple studies (223–225). Focal introduction of histamine or histidine within the central nervous system increases seizure threshold in animal seizure models (Level 5) (224, 226–228). Carnosine has been shown to have an anticonvulsant effect in several animal models of seizures (Level 5) (228–231), thus it receives a GOR D – BR for evidence based on bench research. l-Carnosine has not been evaluated in children with ASD and seizures, so it receives a GOR of N. l-Carnosine appears well tolerated and has the potential to improve both ASD symptoms and seizures, so it may be a promising therapy for individuals with ASD and seizures.

### Omega-3 fatty acids

Several studies have demonstrated a protective effect of omega-3 fatty acids, particularly, docosahexaenoic acid, in animal models of epilepsy (232), creating enthusiasm for their use in humans, especially because of their positive effect on cardiovascular health (233). An early DBPC study (Level 1b) suggested that the effect of omega-3 fatty acids on seizures was transient (234) while more recent small open-label (235) and DBPC (236) studies (Level 2b) suggest a trend toward seizure improvement in individuals with refractory epilepsy. However, another DBPC study (Level 2b) did not confirm this finding (237) in refractory epilepsy. Interestingly, one small DBPC study (Level 2b) in individuals with refractory epilepsy demonstrated significant improvements in parameters of cardiovascular health, particularly in improvement in an index of sudden unexplained death in epilepsy (236) while another small DBPC study (Level 2b) of patients with refractory epilepsy that used magnetic resonance spectroscopy demonstrated a reduction in membrane phospholipid breakdown and improvement in energy metabolism in the brain (238). Thus, omega-3 fatty acids receive a GOR of C for the treatment of epilepsy.

Several studies have noted that essential fatty acids are abnormal in individuals with ASD (239–242). Several small clinical studies have examined the effect of omega-3 fatty acids in children and adults with ASD. Two open-label studies (Level 2b) demonstrated improvements in ASD symptoms (243, 244) while no significant effects could be found in other open-label studies (Level 2b) on adults or children with ASD (245, 246). Two DBPC studies (Level 2b) demonstrated non-significant behavioral improvements in hyperactivity (247, 248) and stereotyped behavior (247) in individuals with ASD. Thus, the evidence for use of omega-3 fatty acids in ASD remains promising, resulting in a GOR of B. Omega-3 fatty acids have not been evaluated in children with ASD and seizures, so it receives a GOR of N. However, it remains a promising novel treatment.

### Homeopathy

Homeopathy uses extremely dilute solutions of active agents to stimulate immune defenses and normalize homeostatic mechanisms. In contrast, the allopathic approach uses high doses of agents to directly attack microorganisms or to block systemic reactions. Homeopathy is viewed skeptically because the main ingredients in its remedies are diluted out of material existence. For example, homeopathic remedies are diluted, at times, beyond Avogadro’s limit, making it unlikely that even one molecule of the agent is in solution. Analysis of homeopathic agents showed both quantitative and qualitative differences from controls using a variety of scientific measures, including ultraviolet spectroscopy, Raman spectroscopy, and thermodynamics (249, 250). These data are used to support the controversial hypothesis that succussion, a process used in the preparation of homeopathic remedies, potentiates biological activity of the solution by generating surrogate structured water domains with biologic activity (49, 251). While older meta-analyses conclude that the clinical effects of homeopathy could not be completely accounted for by a placebo effect (252, 253), a recent meta-analysis that compared homeopathic and allopathic placebo-controlled trials suggested that the weak effects found in homeopathy studies is consistent with a placebo effect (254).

Evidence for the effectiveness of homeopathy treatment for attention-deficit disorder has been documented in a randomized DBPC cross-over trial (Level 1b) (255) suggesting an application to children with ASD. Although several case reports (Level 4) have suggested that homeopathy therapy could be useful in the treatment of epilepsy (256, 257), inconsistency in the homeopathic compounds used and the lack of a temporal relationship between the initiation of homeopathy treatment and the resolution of seizures in these reports limits the strength of this evidence. In addition, the only homeopathy study in ASD reported worsening of behavior with treatment (258). Thus, it receives a GOR of N for epilepsy as the current case reports are too low in quality to conclude anything in particular and a GOR of D – PD for possibly being detrimental in ASD. Since it has not been studied in individuals with ASD and seizures, it receives a GOR of N. Thus, homeopathy does not appear to be a promising treatment for epilepsy and may be detrimental in ASD.

### Diet

Many children with ASD are treated with various diets, including the gluten-free casein-free (GFCF) and elimination diets such as the Feingold diet. Each will be discussed separately.

The GFCF diet has received considerable attention in ASD treatment. A recent two-stage 24-month randomized single-blind controlled trial (Level 1b) has provided support for the GFCF diet (259), yet a relatively brief (12-weeks) DBPC cross-over treatment trial (Level 1b) of the GFCF diet did not demonstrate evidence of objective efficacy despite parental reports of effectiveness (260). In a retrospective, controlled survey study (Level 3b) children with gastrointestinal symptoms, food allergies, and/or sensitivities or those who strictly adhered to the diet, parents rated the GFCF diet as significantly improving ASD behaviors, physiological symptoms, and social behaviors as compared to children without these disorders or in which the diet was implemented with many infractions (261). Thus, for ASD symptoms the GFCF diet receives a GOR of B.

A recent case report (Level 4) demonstrated that the GFCF diet combined with the KD significantly improved seizures in a 12-year-old girl with ASD (44). In a recent case-control survey (Level 3b) of treatments for seizures in children with ASD, parents rated the GFCF diet as a treatment with favorable effects on seizures and other symptoms (Figure 2) (10). The GFCF diet has not been evaluated for epilepsy in individuals without ASD although one report suggests that gluten sensitivity is associated with hippocampal sclerosis in temporal lobe epilepsy (262). Thus, for the treatment of seizures, the GFCF diet receives a GOR of C for ASD individuals and GOR of N for individuals without ASD. One interesting connection between the GFCF diet and seizures is that the GFCF diet is a milk-free diet and a milk-free diet can reduce the serum titer of the FRα autoantibody (see Abnormalities in Folate Metabolism).

The Feingold diet eliminates food additives and therefore is believed to reduce the excitatory-inhibitory balance of the brain (263). A recent systematic review and meta-analysis suggests that artificial food coloring exclusion could have a role in the treatment of attention-deficit hyperactivity disorder (Level 1a) (264), thus supporting the role of the Feingold diet in some neurodevelopmental disorders. The Feingold diet was shown to improve seizures using a single-subject design in a child with TSC not reported to have ASD (Level 4) (263) but has not undergone any additional study on individuals with seizures, epilepsy, or ASD. Thus, the Feingold diet receives a GOR of D – SC for seizures and GOR of N for individuals with ASD with or without seizures.

Clearly, additional controlled clinical trials are needed to document the efficacy of these promising areas of dietary intervention. Dietary interventions are an important area of research, especially since many parents implement dietary intervention on their own and little objective evidence is available to provide parents guidance on these diets.

### Transcranial magnetic stimulation

**Figure 3:**
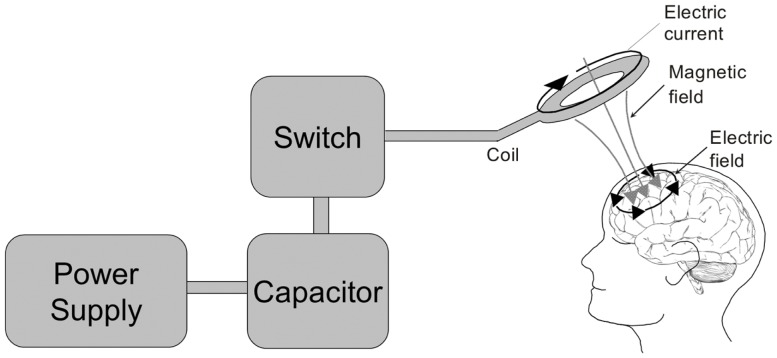
**The transcranial magnetic stimulator**. A block diagram depicting the transcranial magnetic stimulator circuit is depicted on the left. The power supply charges the capacitor. An operator or computer then signals for the charge stored in the capacitor to be released into the stimulation coil through a thyristor switch. The current flowing through the stimulating coil (here depicted as a circular coil) produces a perpendicular magnetic field which transverses the skull and induces electrical currents within the cortex underlying the coil.

Repetitive transcranial magnetic stimulation (rTMS) is a non-invasive technique for manipulating the electrophysiological activity of the cortex and has potential for the study, diagnosis, and treatment of ASD and seizures (Figure 3) (265). Low-frequency (“slow”) rTMS (in the 0.3- to 1-Hz frequency range) which preferentially activates radially oriented, double-bouquet axons, and other inhibitory interneuronal elements has been proposed to operate via long-term depotentiation and depression of cortical activity (266). It is suggested that the application of slow rTMS over the dorsolateral prefrontal cortex of ASD patients may strengthen the inhibitory surrounding minicolumns in this cortical area (267, 268). The rational for using rTMS is based on studies suggesting a minicolumnopathy in ASD resulting in a deficit in cortical inhibition (269, 270). Although rTMS has never been used to treat seizures or epilepsy in ASD, there are several studies that have examined its use in the treatment of ASD symptoms and in the treatment of seizures separately. In individuals with ASD, low-frequency rTMS has been shown to improve error monitoring (271), event-related gamma (267), and repetitive-ritualistic behaviors (267) in two small open-label controlled trials (Level 2b) and to improve event-related potentials related to novelty stimuli in a small open-label uncontrolled trial (Level 2b) (268). Thus, it receives a GOR of B for the treatment of ASD. Three randomized, DPBC studies have examined the effect of low-frequency rTMS for the treatment of epilepsy in adult patients (Level 2b). While only one study demonstrated a significant reduction in seizures (272) another study demonstrated borderline significance for seizure reduction (273), while the third demonstrated a significant decrease in epileptiform abnormalities in one-third of the patients (274). A decrease in epileptiform discharges using low-frequency rTMS was confirmed in another more recent case series (Level 4) (275). Thus, it receives a GOR of B for the treatment of epilepsy. Since slow rTMS has not been studied in individuals with ASD and epilepsy, it receives a GOR of N. Clearly low-frequency rTMS is a promising novel treatment for both epilepsy and ASD and should undergo larger DPBC studies to further investigate its therapeutic effect.

## Summary

This review aimed to outline the evidence for the use of traditional and novel treatments for seizures in individuals with ASD. Using a standardized scale, Table 3 presents the evidence for using these treatments to improve seizures in general, to improve the behavioral and cognitive symptoms associated with ASD, and to improve seizures specifically in individuals with ASD. One of the most obvious conclusions from this review is that few treatments for seizures have been specifically evaluated in individuals with ASD. Specific genetic (93), metabolic (276), and immunologic (276) abnormalities as well as cortical hyperexcitability (277) are believed to be associated with ASD. All of these abnormalities could also drive the development of seizures and epilepsy in ASD individuals. Thus, it is important to determine whether specific treatments that address these pathophysiological mechanisms are effective treatments, as such treatments may improve seizures while also improving, or at least not worsen, core symptoms of ASD.

### Anti-epileptic drug treatment for seizures in autism spectrum disorder

There are no well-controlled clinical trials which examine the effectiveness or efficacy of AEDs for the treatment of seizures in individuals with ASD, despite the fact that this issue has been pointed out almost a decade ago (278). There is good reason to believe that certain AEDs might be more appropriate for certain individuals with ASD. AEDs can cause neurological adverse effects (e.g., ataxia, tremor, and nystagmus), gastrointestinal adverse effects (e.g., abdominal pain and nausea), and allergic reactions. Since adverse effects of AEDs tend to be more frequent in children with developmental disabilities, AEDs with greater risk of adverse effects might limit their usefulness in treating children with ASD (279). In addition, prolonged treatment with AEDs, especially older AEDs, can cause memory and/or attention deficits as well as somnolence, psychomotor abnormalities, and dizziness (280). Alternatively, ASD may be associated with cortical hyperexcitability, potentially due to deficits in cortical inhibitory circuits or glutamate receptor abnormalities (277, 281, 282). Thus, AEDs that enhance γ-aminobutyric acid (GABA) signaling, such as valproate, gabapentin, clobazam, clonazepam, phenobarbital, primidone, tiagabine, or vigabatrin, might be relatively better treatments for individuals with ASD. Lastly, since individuals with ASD appear to have a wide range of seizure types, broad-spectrum AEDs, such as valproate, lamotrigine, or levetiracetam might be optimal treatments.

From the above literature review, it appears that there is only limited evidence to guide the selection of specific AEDs for treating seizures in ASD, although the limited evidence does appear to be consistent across studies. The majority of studies on AEDs in ASD have reported data primarily on three AEDs, valproate, lamotrigine, and levetiracetam. Some studies have also reported the use of phenytoin, carbamazepine, ethosuximide, topiramate, oxcarbazepine, gabapentin, and phenobarbital in individuals with ASD. Overall, studies suggest that broad-spectrum AEDs, specifically valproate, lamotrigine, and levetiracetam, appear to be the most effective and have a low rate of cognitive and neurological adverse effects. Therefore, these may be the most appropriate primary AEDs to start in individuals with ASD and clinical seizures. We will briefly discuss these AEDs below.

Valproate is a broad-spectrum AED which has been documented to have positive behavioral effects in several DBPC studies, has been associated with improvement in core ASD symptoms in case reports and in an open-label trial and, at therapeutic doses, has little adverse effects on cognition (283). Yet it requires multiple blood tests, can deplete carnitine and can result in serious adverse effects, including hepatotoxicity, hyperammonemia, and pancreatitis. l-Carnitine has been shown to decrease the hyperammonemia in patients with valproate-induced encephalopathy and is recommended in severe valproate poisoning. Co-treatment with l-carnitine is recommended for high-risk pediatric patients receiving valproate (284, 285). Given that children with ASD are at high risk for mitochondrial dysfunction and, as a group, generally have low carnitine levels (107, 286), and that carnitine deficiency may be a risk factor for developing valproate hepatotoxicity (287, 288), it is probably wise to start l-carnitine supplementation when prescribing valproate to individuals with ASD especially if they consume little beef or pork, the primary dietary sources of carnitine. The recommended oral dose varies from 30 to 100 mg/kg/day in two to three daily divided doses for high-risk individuals such as young children or those who have carnitine deficiency (284). Thus, it is probably wise to use care when prescribing valproate in children with ASD and monitor carnitine levels during treatment. However, the fact that valproate is reported to be well tolerated and considerably beneficial in many studies examining the ASD population specifically is reassuring.

Lamotrigine is another broad-spectrum AED that is believed to have minimally adverse effects on cognition in individuals with epilepsy (36) and may have beneficial effects on core ASD symptoms (17), or at least not worsen behavior or ASD symptoms (35). The use of lamotrigine as a first-line AED needs to be balanced with the prolonged time required for titration to a therapeutic dose in order to minimize the unlikely but serious adverse effect of a Stevens–Johnson reaction.

Levetiracetam is a relatively broad-spectrum AED that has minimal liver metabolism with a low incidence of serious adverse effects. The most common adverse effect of levetiracetam in the general population is behavioral, including agitation, aggressive behavior, and mood instability. However, it is reassuring that a DBPC trial suggests that there is no change in aberrant or repetitive behaviors or impulsivity or hyperactivity in children with ASD treated with levetiracetam (34). If behavioral adverse effects arise, they may be mitigated with pyridoxine. Major et al. (289) treated patients on an average dose of 63 mg/kg/day of levetiracetam with an average of 6 mg/kg/day of pyridoxine if behavioral problems developed after starting levetiracetam. Of the 22 patients treated, 41% improved, 36% demonstrated no change, and 18% became worse. Improvement in levetiracetam associated behavior abnormalities with pyridoxine treatment was also reported in one case study (290). Since pyridoxine clearly does not work in all cases and since levetiracetam does not appear to cause significant behavioral problems in ASD in controlled studies (34), it is probably best to use pyridoxine on an as needed basis. It is also important to be aware that many children with ASD may already be on a form of pyridoxine as a novel therapy (10). In addition, this review suggests that the combination of pyridoxine with Mg may be more effective for behavior in children with ASD than either one alone, so it may be wise to add Mg to pyridoxine treatment. Levetiracetam has an intravenous and liquid formulation, so it can be titrated quickly, provided to individuals that cannot take medication orally, and given to children who cannot swallow pills.

Mitochondrial disease and dysfunction are prevalent in individuals with ASD (106, 107, 276, 286), and certain AEDs may be more appropriate for individuals with mitochondrial abnormalities. Some worry about the use of valproate in ASD given its potentially devastating effect on children with specific MDs. It is important to understand that this detrimental effect is isolated to individuals with POLG1 mutations and myoclonic epilepsy with ragged red fibers syndrome (291). POLG1 mutations have only been reported in two children with ASD, or 2% of ASD children reported to have MD, and myoclonic epilepsy with ragged red fibers has not been reported to date in individuals with ASD (107). Given that approximately 5% of children with ASD have classically defined MD, the estimated prevalence of the POLG1 mutation is therefore approximately 0.1% of the ASD population. One study has examined the effect of common AEDs, including phenobarbital, carbamazepine, and lamotrigine, on mitochondrial function. While carbamazepine showed a detrimental effect on mitochondrial function with chronic use, lamotrigine was found to enhance mitochondrial function (292). Other *in vitro* studies have also demonstrated that lamotrigine is mitochondrial protective (293). Thus, there is little evidence to guide the understanding of optimal AED treatments for children with mitochondrial abnormalities with and without ASD. However, given that lamotrigine appears to be well tolerated in individuals with ASD and may have positive effects on mitochondrial function, at least in preliminary studies, lamotrigine may be the optimal AED for children with ASD and MD. This is indeed a ripe area for clinical research.

Overall, the data reviewed above supports the use of valproate, lamotrigine, and levetiracetam as the first-line treatments in children with ASD who have seizures or epilepsy. Table 4 outlines some guidelines that might be helpful for choosing first-line AEDs. Clearly more research is needed to document the efficacy of AEDs in the ASD population.

**Table 4:** **Guidelines for selecting a first-line antiepileptic drug for children with autism spectrum disorder**.

ASD symptoms	Avoid	Possible alternative
Gastrointestinal disorders	Valproate	Levetiracetam, lamotrigine
Mitochondrial disorders	Valproate	Levetiracetam, lamotrigine
Poor growth	Topiramate	Lamotrigine
Overweight	Valproate	Lamotrigine, levetiracetam
Behavioral problems	Levetiracetam	Valproate

### Traditional non-anti-epileptic drug treatment for seizures in autism spectrum disorder

Several traditional non-AED treatments for seizures were reviewed, specifically the KD and MAD, the VNS, standard epilepsy surgery and MST, immunomodulatory therapy, and neurofeedback. Overall, many of these therapies, except for standard epilepsy therapy (i.e., cortical resection) and the VNS, appear to have promising applications for the treatment of seizures in children with ASD.

The KD and MAD may be useful for treating several aspects of ASD, especially in individuals with ASD with seizures and/or MD. Indeed, recently there has been increased interest in using these diets in ASD (294–297). The KD may be an effective treatment for MD (298–300) and has been recommended for individuals with co-occurring MD and epilepsy (301). Given that individuals with ASD and co-occurring MD have high rates of seizures (107), the KD and MAD should be strongly considered in the subgroup of individuals with ASD and co-occurring MD. In addition, given the excellent safety profile of the KD and MAD as well as studies which suggest their effectiveness in drug-resistant epilepsy and their tolerability in ASD, the KD and MAD should be considered in children with ASD who have epilepsy that is refractory to standard treatments. Of course, children should be carefully monitored when the KD is started as the diet can worsen the metabolic acidosis associated with mitochondrial or other metabolic disorders and should be managed by a practitioner experienced with these diets.

Although standard epilepsy surgery may be helpful for controlling seizures, there does not appear to be good evidence supporting the notion that standard epilepsy surgery improves cognition or symptoms associated with ASD, and there are many cases in which standard epilepsy surgery has worsened these factors. Several case series have suggested that MST may improve both seizures and ASD related symptoms. Impressive outcomes have been demonstrated in one study in which children underwent extensive electrophysiological study and had careful surgically treated foci with MST. However, MST requires more extensive study before it can be routinely used as a treatment option for children with ASD who have refractory epilepsy.

Given the growing literature on immune dysregulation in ASD (276), it would not be surprising if immunomodulatory treatments would be useful in children with ASD and seizures. There are promising studies for the use of both IVIG and steroids for the treatment of epilepsy, ASD related symptoms, and seizures in individuals with ASD, although none of these studies are high quality. Given that these treatments target specific pathophysiological mechanisms, the development of clear guidelines for identifying children with ASD with or without epilepsy who might benefit from such treatment would be helpful, especially given the potential adverse effect of long-term use of immunomodulatory therapies such as steroids. Clearly this is an area that is ripe for further clinical study.

Neurofeedback is an interesting emerging treatment for both seizures and ASD symptoms that has an excellent safety profile and has growing evidence for its effectiveness. However, studies have been limited to particular individuals who could cooperate with the treatment protocol and have underrepresented very young children and adults as well as lower functioning individuals and those with more severe ASD symptoms. Because of the safety of neurofeedback, this may be a promising treatment for children with ASD but further blinded studies would strengthen the evidence of effectiveness and efficacy for this therapy.

Thus, several non-AED traditional therapies demonstrate considerable promise in the treatment of seizures in individuals with ASD, including low-carbohydrate diets such as the KD and MAD, MST, immunomodulatory therapy, and neurofeedback, although more research is needed in all of these areas to gain better evidence for the efficacy and effectiveness of these therapies as well as which specific ASD subgroups might best respond to these therapies. Standard epilepsy surgery therapy raises the risk of making ASD symptoms worse and the VNS has not been shown to improve ASD symptoms in several studies. Thus, these latter two therapies should probably be reserved for epilepsy that is refractory to other epilepsy treatments.

### Treatments for seizures in genetic and metabolic syndromes associated with autism spectrum disorder

This review examined treatments for specific syndromes associated with ASD and seizures, including genetic syndromes such as TSC and Fragile X and metabolic disorders such as mitochondrial disease and dysfunction, urea cycle disorder, succinic semialdehyde dehydrogenase, branched-chain ketoacid dehydrogenase kinase, creatine and biotinidase deficiency, Smith–Lemli–Opitz syndrome, pyridoxine-dependent and responsive seizures, organic acidemias, and abnormalities of folate and cobalamin metabolism.

Mitochondrial disease and dysfunction and abnormalities in cerebral folate metabolism are two metabolic abnormalities that are associated with seizures and appear to affect a substantial portion of individuals with ASD. Several therapies for these two disorders have demonstrated effectiveness in the treatment of symptoms associated with ASD in controlled studies, particular l-carnitine, multivitamins with antioxidants, *N*-acetyl-l-cysteine, and folinic acid. Folinic acid has also been shown to improve seizures in individuals with seizures and cerebral folate abnormalities in uncontrolled studies. Thus, these treatments may be very useful for some individuals with ASD and seizures and should be strongly considered in selected cases.

Evidence also exists for a defect is cobalamin associated metabolism in ASD although not for a cobalamin deficiency itself. Still there is limited evidence that methylcobalamin can improve ASD symptoms and glutathione metabolism in children with ASD and other evidence that cobalamin supplementation can be useful in seizures due to cobalamin deficiency. Because methylcobalamin appears to be potentially useful for individuals with ASD, this treatment could be useful for those with ASD and seizures, but further research is needed before such recommendations can be made.

Several case studies have suggested that ASD can occur in specific metabolic syndromes that have specific treatment. In limited studies, within the context of the specific syndrome, treatments for creatine deficiency, d-glyceric aciduria and pyridoxine-dependent and responsive seizures appear to be helpful for treating seizures, while treatments for creatine deficiency, d-glyceric aciduria, and urea cycle disorders appear to be helpful for ASD associated symptoms. Treatments for biotinidase deficiency, branched-chain ketoacid dehydrogenase kinase deficiency, and semialdehyde dehydrogenase deficiency do not appear to be helpful for ASD symptoms or seizures in individuals with ASD, but this is based on limited reports.

For the two genetic syndromes reviewed, there are no clinically tested treatments that address the underlying pathophysiological mechanisms believed to be involved, although bench research is actively ongoing. For TSC, vigabatrin is the AED of choice for infantile spasms and seizures starting in infancy and appears to be most effective when started early in life. For Fragile X, standard AED therapy appears to be the mainstay at this time. Hopefully ongoing clinical trials will provide evidence for novel efficacious therapies that address the underlying pathophysiology of these syndromes.

This review provides guidance for treatment of seizures in ASD for specific syndromes and suggests that certain novel treatments, including l-carnitine, multivitamins with antioxidants, *N*-acetyl-l-cysteine, and folinic acid, may be helpful in a wider number of individuals with ASD and seizures.

### Potential novel treatments for seizures in autism spectrum disorder

This review examined novel treatments that may have potential use for individuals with ASD and seizures. Such novel treatments included Mg, pyridoxine and Mg combined, Zn, DMG, taurine, l-carnosine, omega-3 fatty acids, homeopathy, the GFCF and Feingold/elimination diets, and low-frequency repetitive transcranial magnetic stimulation.

Mg has good evidence as an adjunctive therapy for seizures but does not have evidence to support its use for ASD associated symptoms. However, there is evidence that Mg combined with pyridoxine can improve ASD associated symptoms. As neither pyridoxine nor Mg alone appear to be detrimental to ASD associated symptoms and pyridoxine itself can be a treatment for underlying metabolic deficiencies, the combination of Mg and pyridoxine for treatment of individuals with ASD and seizures could be a good treatment to investigate. For an adjunctive treatment for individuals with ASD and seizures, Mg is a good candidate, especially since it can also treat constipation which is a symptom commonly seen in individuals with ASD, particularly those with mitochondrial abnormalities.

There has been significant interest in the use of omega-3 fatty acids for epilepsy and for ASD. Preliminary reports are encouraging but it is clear that the effects are subtle and require larger clinical samples to obtain statistically significance in clinical studies. One interesting aspect of this supplement is its potential cardiovascular benefits in epilepsy patients, specifically improvement in the index of sudden unexplained death in epilepsy. This is particularly important as individuals with ASD and epilepsy appear to have higher rates of unexplained mortality than individuals with ASD without epilepsy (302).

The GFCF diet appears to be useful in some children with ASD and has been rated as improving seizures in a controlled survey study of ASD patients with seizures. Interestingly, a recent case report has suggested that combining the GFCF diet with the KD may have some utility in refractory epilepsy in ASD. The GFCF diet is also a milk-free diet which can decrease the folate-receptor alpha autoantibody. Thus, the GFCF diet could have some utility for seizure control in families that are able to implement it, although controlled studies are clearly needed to determine this possibility.

l-Carnosine has evidence of usefulness in the treatment of ASD associated symptoms and bench research suggests that carnosine and its active metabolite may be therapeutic in animal models of epilepsy. Thus, l-carnosine might be a novel treatment that has usefulness in the treatment of seizures in individuals with ASD. In addition, Zn has been shown in bench research to modulate neuroexcitability and in clinical studies has been found to be abnormal in individuals with seizures. As Zn abnormalities have been described in individuals with ASD, this may be a novel treatment for individuals with ASD and seizures.

Transcranial magnetic stimulation is an emerging treatment that has shown efficacy in both seizures and ASD symptoms in limited studies and appears to be safe in children. Although this treatment requires a relatively cooperative patient, thus limiting the specific ASD patients in which it is applicable, it might be a beneficial therapy for selected patients with refractory epilepsy. Clearly more research and high-quality clinical trials would be useful to further investigate this therapy.

Certain treatments, such as taurine and homeopathy, have the potential to be detrimental, at least based on limited evidence, thus suggesting that they should be generally avoided in individuals with ASD and seizures. In limited studies, DMG does not appear to be useful for treatment of seizures or ASD symptoms, so it will probably not be generally useful for the treatment of seizures in ASD.

Thus, this review suggests that certain novel treatments, such as Mg with pyridoxine, omega-3 fatty acids, the GFCF diet, and low-frequency repetitive transcranial magnetic simulation could be useful for the treatment of seizures and ASD symptoms while other treatments such as Zn and l-carnosine hold some promise. Other treatments such as taurine, DMG, and homeopathy are probably unlikely to be of use.

### Limitations of previous studies and guidelines for future studies

Many of the studies reviewed have substantial limitations, particularly in their approach to documenting the effectiveness of treatments, defining ASD and following improvement in seizures and ASD symptoms. Few studies are high-quality, controlled, and/or blinded, and even the high-quality studies have relatively small populations thus limiting the generalizability of the findings. Many studies do not have well-defined populations and do not use standardized tools such as the Autism Diagnostic Observation Schedule or the Autism Diagnostic Interview – Revised to document the diagnosis of ASD, and other important factors such as language and intellectual development are not clearly defined in most studies. Furthermore, many studies do not quantitatively measure changes in seizures and many studies use various criteria to define seizure improvement. There is also a great need for long-term studies, since many of the treatments are used for years or even decades, whereas most clinical studies are usually months in duration, and some of the medications may have long-term adverse effects. Thus, in the future, promising treatments for seizures in individuals with ASD must be evaluated in high-quality DBPC studies in order to establish effectiveness, with long-term follow-up.

## Conclusion: An Approach to Treating Children with ASD for Seizures

There are currently no guidelines for the treatment of seizures in individuals with ASD. Seizures can be treated by a variety of approaches. Determining the underlying medical etiology of seizures will assist in directing specific treatment. Many individuals with ASD and seizures could manifest mitochondrial disease or dysfunction or cerebral folate abnormalities. Identifying and treating such underlying abnormalities could provide substantial benefit to the patients and improve seizures. For etiologies without specific treatments or when an etiological cause cannot be identified, there appears to be specific AEDs that may be most promising as first-line treatments, particularly lamotrigine, valproate, and levetiracetam. Of course, patient characteristics should guide the AED choice as well as adjunctive treatments to minimize adverse effects. Promising non-AED treatments, such as the KD and MAD, may also be particularly useful in ASD individuals with epilepsy. Some novel treatments such as Mg could theoretically help treat seizures as adjunctive treatments but they require critical study in order to be considered for general use in the treatment of seizures in children with ASD.

## Authors Contribution

Portions of this manuscript were developed during the Elias Tembenis Seizure Think Tanks at the AutismOne Meeting in Chicago in May of 2009 and 2010 and at the AutismOne Canada Meeting in Toronto, ON, Canada in October of 2009. These Think Tanks included scientists and clinicians with expertise in seizures related to ASD. The participants represented a wide variety of researchers and practitioners who treat ASD. The participants from the initial Think Tank in May of 2009 provided the basis for the content of the information within this manuscript. Individuals in the following two Think Tanks (October 2009 and May 2010) provided suggestions for the developed document. Individual participants who provided written text for the supplement or contributed in the editing of the document are recognized as authors. Dr. Fatemi’s research fellow assisted with the writing but did not attend the Think Tanks.

## Conflict of Interest Statement

The authors declare that the research was conducted in the absence of any commercial or financial relationships that could be construed as a potential conflict of interest.

## Abbreviations

5MTHF, 5-methyltetrahydrofolate; AEDs, anti-epileptic drugs; AER, autistic epileptiform regression; ASD, autism spectrum disorder; BR, bench research; Ca, calcium; DBPC, double-blind placebo-controlled; PD, possibly detrimental; DMG, dimethylglycine; EEG, electroencephalogram; FRα, folate-receptor alpha; GABA, γ-aminobutyric acid; GFCF, gluten-free casein-free; GOR, grade of recommendation; HEADD, hypotonia, epilepsy, autism, and developmental delay; IVIG, intravenous immunoglobulin; KD, ketogenic diet; MAD, modified Atkins diet; MD, mitochondrial disease; MEG, magnetoencephalography; Mg, magnesium; MgSO_4_, Mg sulfate; MST, multiple subpial transections; N, no studies; NE, neutral effect; rTMS, repetitive transcranial magnetic stimulation; SC, single case report or series; TSC, tuberous sclerosis complex; VNS, vagal nerve stimulator; Zn, zinc.
